# Die hausärztliche Versorgung von Menschen mit Behinderungen in München: Ergebnisse einer Querschnittstudie

**DOI:** 10.1055/a-2833-6852

**Published:** 2026-06-22

**Authors:** Antonia Pflueger, Lukas Schweppe, Michaela Coenen

**Affiliations:** 1 Ambulante und klinische Strukturen, Gesundheitsreferat München, MünchenGermany; 2 Institut für Medizinische Informationsverarbeitung, Biometrie und Epidemiologie, Lehrstuhl für Public Health und Versorgungsforschung, Medizinische Fakultät, Ludwig-Maximilians-Universität München, MünchenGermany; 3 Fachstelle Inklusion und Gesundheit, Gesundheitsreferat München, MünchenGermany; 4 Pettenkofer School of Public Health, MünchenGermany

**Keywords:** medizinische Versorgung, ambulant, Beeinträchtigungen, Behindertenhilfe, Barrieren, Inanspruchnahme, medical care, outpatient, impairments, institutions for people with disabilities, barriers, utilization of medical services

## Abstract

**Hintergrund:**

Trotz gesetzlicher Vorgaben zur diskriminierungsfreien ärztlichen Versorgung empfinden Menschen mit Behinderungen (MmB) ihre hausärztliche Versorgung häufig als unzureichend. Sie berichten von Barrieren beim Zugang zur Versorgung und Unsicherheiten des medizinischen Personals. Während Kinder in sozialpädiatrischen Zentren betreut werden, gibt es für Erwachsene mit Behinderungen keine Alternativen. Zwar etablieren sich Medizinische Zentren für Erwachsene mit Behinderungen, allerdings sind diese nicht dafür gedacht, Mängel in der ambulanten Versorgung von MmB abzufangen. Ziel dieser Studie war es, die hausärztliche Versorgungssituation von MmB aus Sicht der MmB in München zu untersuchen.

**Methodik:**

In einer Querschnittstudie wurden MmB ohne Ausschlusskriterien in München mittels eines Fragebogens von August bis Oktober 2024 zu ihrer hausärztlichen Versorgung befragt. Die Befragung konnte in Alltags- und leichter Sprache online und schriftlich durchgeführt werden. Mit Hilfe deskriptiver Statistik und bivariater Analysen wurden Häufigkeiten und Zusammenhänge untersucht. Durch eine logistische Regressionsanalyse wurden Prädiktoren für die Wahrscheinlichkeit, Schwierigkeiten bei der Suche nach einem Hausarzt zu haben oder gehabt zu haben berechnet.

**Ergebnisse:**

An der Befragung nahmen 306 Personen teil. Davon gaben 95% (n=291) an, eine Hausärztin/ einen Hausarzt zu haben und über 80% waren mit ärztlichem (n=253) bzw. Praxispersonal (n=252) zufrieden. Dennoch berichteten 39% (n=111) von Schwierigkeiten bei der Ärztin-/Arztsuche und 33% (n=91) wurden schon einmal von einer Praxis abgewiesen. Es zeigten sich Unterschiede in der Versorgung hinsichtlich der Inanspruchnahme von Terminen, der Notwendigkeit des Besuches des Hausarztes/ der Hausärztin aufgrund der Beeinträchtigung, Schwierigkeiten bei der Suche nach einer ärztlichen Praxis sowie bei der freien Ärztin/Arztwahl und der Aufnahme in eine hausärztliche Praxis, in Abhängigkeit von Geschlecht, Wohnform und Art der Beeinträchtigung sowie bei der Notwendigkeit von Hausbesuchen. Zudem ergaben die multivariaten Analysen, dass MmB ein signifikant höheres Risiko für Schwierigkeiten bei der Suche nach einer Hausärztin/ einem Hausarzt haben, wenn sie schon einmal von einer Praxis abgewiesen wurden, beim Arztbesuch eingeschränkt sind oder Hausbesuche benötigen.

**Schlussfolgerung:**

Die Studie zeigt, dass die hausärztliche Versorgung aus Sicht der MmB als zufriedenstellend eingestuft wurde, jedoch Zugangsprobleme aufgrund baulicher, sprachlicher sowie sozialer Barrieren bestehen. Sie bietet erste Ergebnisse und Hinweise auf daraus entstehende Herausforderungen bei Hausarztbesuchen. Die Ergebnisse können dazu beitragen, Akteur*innen praxisnahe Argumente für eine Verbesserung der Versorgungssituation zu liefern.

## Abstract (en)

**Background:**

Despite laws for non-discriminatory medical care, people with disabilities often perceive their primary care as insufficient. They report barriers to access and uncertainties among medical staff. The aim of this study was to examine the primary care situation for people with disabilities in Munich.

**Methods:**

In this cross-sectional study, between August and October 2024, people with disabilities in Munich were surveyed about their primary care experience using a questionnaire. No exclusion criteria were applied. The survey was conducted both online and in written form, using plain- and easy-to-read language. Data were analyzed using descriptive statistics, and bivariate analyses were conducted to examine associations. Logistic regression analysis was used to calculate predictors for the likelihood of having or having had difficulties in finding a general practitioner.

**Results:**

A total of 306 people participated in the survey; 95% (n=291) reported having a GP, and over 80% were satisfied with medical (n=253) and practice staff (n=252). Nevertheless, 39% (n=111) reported difficulties in finding a GP, and 33% (n=91) had been refused by a practice at least once. Differences in care were observed regarding the utilization of appointments, the need to visit a GP due to disability, difficulties in finding a medical practice, freedom of choice of physician, and acceptance into a GP practice, depending on gender, living situation, and type of disability, as well as with regard to the need for home visits. In addition, multivariate analyses showed that people with disabilities had a significantly higher risk of experiencing difficulties in finding a GP if they had previously been refused by a practice, were restricted during medical visits, or needed home visits.

**Conclusion:**

The study shows that, from the perspective of people with disabilities, primary care was rated as satisfactory; however, access problems persist due to structural, communication and social barriers. The results can help provide stakeholders with practical arguments to improve the care situation.

## Einleitung

Es ist ein Grundrecht in der Europäischen Union, dass jeder Mensch das Recht auf Zugang zur Gesundheitsvorsorge und auf ärztliche Versorgung hat 1 . Nach der UN-Behindertenrechtskonvention haben Menschen mit Behinderungen (MmB) ein Recht auf das erreichbare Höchstmaß an Gesundheit ohne Diskriminierung aufgrund von Behinderung 2 . Angehörige der Gesundheitsberufe sind verpflichtet, MmB eine Versorgung von gleicher Qualität wie Menschen ohne Behinderungen zu ermöglichen, auch auf Grundlage der freien Einwilligung und nach vorheriger Aufklärung. Eine diskriminierende Vorenthaltung von Gesundheitsversorgung und Gesundheitsleistungen ist nach der UN-Behindertenrechtskonvention zu verhindern 2 .

Trotz dieser gesetzlichen Regelungen ist der Zugang zu gesundheitlichen Versorgungsangeboten für MmB in Deutschland erschwert 3 4 5 6 . Häufig benötigen MmB aufgrund komplexer Krankheitsbilder eine umfassende und fachübergreifende Versorgung 4 6 . Während diese im Kindes- und Jugendalter durch sozialpädiatrische Zentren abgedeckt werden kann, gibt es für MmB im Erwachsenenalter keine vergleichbaren Versorgungsangebote 6 , was unter anderem zu Problemen in der ambulanten Versorgung führt. Zwar sind seit 2015 Medizinische Zentren für Erwachsene mit Behinderungen gesetzlich verankert, allerdings gibt es hierfür hohe Zugangs- und Zulassungsvoraussetzungen 7 und schwierige Vergütungsverhandlungen, was dazu führte, dass deutschlandweit bisher nur 57 Medizinische Zentren für Erwachsene mit Behinderungen betrieben werden (Stand 2023) 8 . Die Bundesarbeitsgemeinschaft der Medizinische Zentren für Erwachsene mit Behinderungen betont, dass Medizinische Zentren für Erwachsene mit Behinderungen nicht dafür gedacht sind, bestehende Mängel des Gesundheitssystems (z. B. fehlende Barrierefreiheit) zu kompensieren, sondern eine Ergänzung für eine zielgruppenspezifische, bedarfsgerechte Versorgung sein sollen.

Die hausärztliche Versorgung nimmt eine zentrale Rolle in der Versorgung von MmB ein. Sie umfasst Gesundheitsvorsorge, Krankheitsfrüherkennung und -behandlung, Rehabilitation, Betreuung bei medizinischer, sozialer und psychischer Hilfsbedürftigkeit sowie die Zusammenarbeit mit Fachärzt*innen und Krankenhäusern 9 . Zur gesundheitlichen Versorgung von MmB gibt es in Deutschland bisher nur geringe Evidenz 3 4 . Zur Identifikation politischer Handlungsbedarfe und Verbesserung der Versorgung von MmB ist diese von großer Relevanz 4 .

Vertreter*innen von Einrichtungen der Behindertenhilfe in München haben das Gesundheitsreferat der Landeshauptstadt München darauf hingewiesen, dass sie die Sicherstellung einer angemessenen hausärztlichen Versorgung für ihre Bewohner*innen mit Behinderungen vor Herausforderungen stellt. Es ist anzunehmen, dass die bedarfsgerechte ambulante hausärztliche Versorgungssituation auch von MmB außerhalb dieser Einrichtungen als eingeschränkt wahrgenommen wird. Die Fachstelle „Inklusion und Gesundheit” des Gesundheitsreferats der Landeshauptstadt München führte daher eine Befragung zur hausärztlichen Versorgung von MmB in München durch. Ziel dieser Studie ist es, die Inanspruchnahme der hausärztlichen Versorgung aus Sicht der MmB in München zu analysieren sowie mögliche Einschränkungen beim Zugang zur Versorgung zu identifizieren.

Spezifische Fragestellungen sind:

1. Welche Aspekte der Barrierefreiheit erfüllen hausärztliche Praxen in München?
2. Wie zufrieden sind MmB mit ihrer Hausärztin/ihrem Hausarzt, dem Praxispersonal und der medizinischen Behandlung?
3. Gibt es Unterschiede in der hausärztlichen Versorgung von MmB in Abhängigkeit von Alter, Geschlecht, Art der Behinderung und Wohnform?
4. Gibt es Unterschiede in der hausärztlichen Versorgung bei MmB mit und ohne Bedarf nach Hausbesuchen?
5. Welche Faktoren beeinflussen die Wahrscheinlichkeit, Schwierigkeiten bei der Suche nach einer Hausärztin/einem Hausarzt zu haben?

## Methodik

### Design

Die Fachstelle „Inklusion und Gesundheit” des Gesundheitsreferats der Landeshauptstadt München führte in Kooperation mit dem Lehrstuhl für Public Health und Versorgungsforschung der Ludwig-Maximilians-Universität München eine anonyme Querschnittstudie zur ambulanten hausärztlichen Versorgungsituation aus der Perspektive von MmB in München durch. Diese Studie ist die erste explorative Studie, die zum Thema hausärztliche Versorgung von MmB in München durchgeführt wurde. Sie wurde von der Ethikkommission der Ludwig-Maximilians-Universität München (Projektnummer 24-0844-KB, 09.10.2024) begutachtet und freigegeben.

### Stichprobe

In die Studie eingeschlossen wurden MmB unabhängig von Beeinträchtigungsform, Diagnosen, Einschränkungen, Grad der Behinderung oder Wohnform. MmB sind laut UN-Behindertenrechtskonvention Menschen, die „langfristige körperliche, seelische, geistige oder Sinnesbeeinträchtigungen haben, welche sie in Wechselwirkung mit verschiedenen Barrieren an der vollen, wirksamen und gleichberechtigten Teilhabe an der Gesellschaft hindern können“ 2 . In der Landeshauptstadt München wohnen 171 257 MmB (Stand 31.12.2023, Grad der Behinderung>20), was etwa 11% der Gesamtbevölkerung in München (1 589 026, Stand 31.12.2023) ausmacht 10 .

Für diese Studie wurden MmB rekrutiert, die entweder ihren Wohnsitz in München hatten oder deren Hausärztin/Hausarzt in München praktizierte. Da die hausärztliche Versorgung in der Regel mit Beginn des Erwachsenenalters die kinderärztliche Versorgung ablöst, wurde das Mindestalter zur Teilnahme an der Befragung auf 18 Jahre festgelegt. Ausschlusskriterien wurden nicht festgesetzt.

### Konzeptentwicklung und Fragebogen

Da es kein etabliertes Erhebungsinstrument zur Erfassung der Inanspruchnahme der hausärztlichen Versorgung von MmB gibt, wurde von der Fachstelle „Inklusion und Gesundheit“des Gesundheitsreferats München in Kooperation mit der Ludwig-Maximilians-Universität in einem iterativen Prozess ein Konzept mit Hintergrund und Zielsetzung der Befragung aufgestellt und anschließend ein Fragebogen entwickelt. Konzept und Fragebogen wurden mit Mitarbeitenden großer Träger der Behindertenhilfe (Stiftung Pfennigparade München, Lebenshilfe München und Gemeinsam Leben Lernen e.V.) besprochen. So sollte sichergestellt werden, dass die Befragung möglichst barrierearm und ressourcenschonend durchgeführt werden konnte. Der Fragebogen wurde in Alltags- und leichter Sprache zur Verfügung gestellt und mit Interessensvertretungen der MmB in München (Behindertenbeauftragter und Behindertenbeirat der Landeshauptstadt München) abgestimmt und pilotiert.

Informationen zum Inhalt des Fragebogens sind in Tab. 1 dargestellt.

**Table:** **Tab. 1** Inhalt des Fragebogens, gegliedert nach fünf inhaltlichen Teilen mit jeweiligen Überschriften in leichter und Alltagssprache, Inhalt, Fragen und Namen der Variablen. Anmerkung: in den Fragebögen wurde auf gendergerechte Sprache verzichtet, weshalb auch in dieser Tabelle aufgrund inhaltlicher Richtigkeit nicht gegendert wird.

Teil	Überschrift	Überschrift leichte Sprache	Anzahl der Fragen	Inhalt	Fragen	Variablen
0	keine	keine	1	Angabe, von wem der Fragebogen ausgefüllt wird: selbständig, mit Unterstützung oder von Angehörigen/ Mitarbeitenden einer Wohneinrichtung oder betreuenden Personen für eine Person mit Beeinträchtigungen	Frage 1: Wer füllt diesen Fragebogen aus?	Frage 1: Angabe zum Ausfüllen
1	Allgemeine Fragen zur hausärztlichen Versorgung	Fragen zu Hausärzten	8	Notwendigkeit einer hausärztlichen Versorgung, Häufigkeit der Inanspruchnahme, fachärztliche Behandlung, Inanspruchnahme/ Notwendigkeit von Hausbesuchen, beeinträchtigungsbedingte Herausforderungen	Frage 2: Haben Sie einen Hausarzt?	Frage 2: Hausarzt vorhanden ^b^
					Frage 2a: Falls ja, haben Sie sich Ihren Hausarzt selbst ausgesucht?	Frage 2a: Hausarzt selbst ausgesucht ^b^
					Frage 2b: Falls ja, benötigen Sie den Hausarzt aufgrund Ihrer Beeinträchtigungen?	Frage 2b: Hausarzt aufgrund von Beeinträchtigungen ^b^
					Frage 3: Hatten/Haben Sie Schwierigkeiten bei der Suche nach einem Hausarzt?	Frage 3: Schwierigkeiten bei der Suche ^b, c, d^
					Frage 4*: Gehen Sie regelmäßig zum Facharzt?	Frage 4: Facharzt vorhanden
					Frage 5: Wann war Ihr letzter Termin beim Hausarzt?	Frage 5: letzter Termin ^b, c^
					Frage 6: Wie häufig hatten Sie in den letzten 12 Monaten einen Termin bei Ihrem Hausarzt?	Frage 6: Termin letzte 12 Monate ^b, c^
					Frage 7: Bietet Ihr Hausarzt Hausbesuche an?	Frage 7: Angebot Hausbesuche
					Frage 8: Benötigen Sie Hausbesuche von Ihrem Hausarzt?	Frage 8: Notwendigkeit Hausbesuche ^b, d^
					Frage 9: Wurden Sie schon mal von einem Hausarzt abgewiesen?	Frage 9: vom Hausarzt abgewiesen ^b, c, d^
2	Unterstützung bei Arztbesuchen	Fragen zur Unterstützung beim Arzt	2	Notwendigkeit der Unterstützung bei Terminvereinbarungen und Begleitung zum Arztbesuch	Frage 10*: Wer kümmert sich um Terminvereinbarungen bei dem Hausarzt?	Frage 10: Terminvereinbarungen
					Frage 11*: Sind Sie auf eine Begleitung zum Hausarzt angewiesen?	Frage 11: Notwendigkeit einer Begleitung
					Frage 11a: Falls ja, haben Sie Schwierigkeiten eine Begleitperson zum Hausarzt zu finden?	Frage 11a: Schwierigkeiten bei Suche nach Begleitung
3	Barrieren beim Arztbesuch	Schwierigkeiten beim Haus-Arzt	2	Einschränkungen beim Arztbesuch, Formen der Barrierefreiheit (baulich, sprachlich, sozial)	Frage 12*: Sind Sie eingeschränkt, wenn Sie zum Hausarzt gehen?	Frage 12: Einschränkungen beim Hausarztbesuch ^b, d^
					Frage 13: Bitte machen Sie Angaben zu Barrierefreiheit bei Ihren Hausarztbesuchen, Abfrage von baulichen, sprachlichen und sozialen Barrieren**	Frage 13: Barrieren
4	Zufriedenheit beim Arztbesuch	Zufriedenheit beim Haus-Arzt	4	Zufriedenheit mit Hausärztin /Hausarzt, Praxispersonal und medizinischer Behandlung	Frage 14: Wie zufrieden sind Sie mit Ihrem Hausarzt?	Frage 14: Zufriedenheit Hausarzt
					Frage 15: Wie zufrieden sind Sie mit dem Praxispersonal in Ihrer Hausarztpraxis?	Frage 15: Zufriedenheit Praxispersonal
					Frage 16: Wie zufrieden sind Sie mit der medizinischen Behandlung beim Hausarzt?	Frage 16: Zufriedenheit medizinische Behandlung
					Frage 17: Fühlen Sie sich mit Ihren Bedürfnissen und Anliegen von Ihrem Hausarzt ernstgenommen?	Frage 17: Ernstgenommen vom Hausarzt
5	Angaben zur Person	Informationen über Sie	7	Alter, Geschlecht, Art der Beeinträchtigung, Grad der Behinderung (GdB), Merkzeichen, Wohnform und Krankenversicherung	Ihr Geschlecht	Geschlecht ^a, d^
					Ihr Alter	Alter ^a, c^
					Welche Beeinträchtigung trifft auf Sie zu?*	Art d. Beeinträchtigung ^a, c, d^
					Haben Sie einen Grad der Behinderung?	GdB
					Falls ja, wie hoch ist Ihr Grad der Behinderung?	Höhe GdB
					Falls Sie einen Grad der Behinderung haben, haben Sie zusätzlich Merkzeichen bekommen?	Merkzeichen erhalten
					Falls ja, welche der genannten Merkzeichen haben Sie erhalten?*	Angaben zu den Merkzeichen
					Welche Wohnform trifft auf Sie zu?	Wohnform ^a, c, d^
					Wie sind Sie krankenversichert?	Krankenversicherung ^d^

*Mehrfachantworten waren möglich, ** Diese Einordnung erfolgte nach den Kriterien für eine barrierefreie Arztpraxis des Robert-Koch-Instituts (RKI) 5 . ^a^ Variable wurde in bivariaten Auswertungen zur hausärztlichen Versorgung berücksichtigt, ^b^ Variable wurde verwendet, um die „hausärztlichen Versorgung“ abzubilden, ^c^ Variable wurde für bivariate Analysen zur Untersuchung der Versorgung bei Bedarf nach Hausbesuchen verwendet, ^d^ Variable wurde umkodiert bzw. kollapsiert aufgrund geringer Ausprägungen

### Datenerhebung

An der Befragung konnte online und in Papierform in leichter und in Alltagssprache teilgenommen werden. Einrichtungen der Behindertenhilfe und Akteur*innen der Behindertenhilfe in München wurden Informationen zum Hintergrund der Befragung, der Fragebogen im PDF-Format sowie Link und QR-Code zur Online-Befragung per E-Mail zugesendet.

An alle Einrichtungen der Eingliederungshilfe in München wurden Papierfragebögen verschickt. Hierfür wurde mittels einer Kontaktdatenliste (Stand Januar 2024) des Gesundheitsreferates München eine Übersicht aller stationärer Einrichtungen der Behindertenhilfe und Wohngruppen in München erstellt und die Anzahl der Wohnplätze ermittelt. Es ist davon auszugehen, dass diese Übersicht vollständig ist und alle stationären Einrichtungen der Behindertenhilfe und Wohngruppen in München enthält. Einrichtungen, in denen mehr als 100 Personen wohnten, erhielten 20 Fragebögen (10 in Alltagssprache, 10 in leichter Sprache), Einrichtungen, in denen mehr als 10 Personen wohnten, erhielten 10 Fragebögen (5 in Alltagssprache, 5 in leichter Sprache). An Einrichtungen, in denen weniger als 10 Personen wohnten, wurde die Anzahl der Fragebögen der Anzahl der Bewohner*innen angepasst. Dieses Vorgehen wurde in Absprache mit den jeweiligen Einrichtungen der Behindertenhilfe vor Versendung der Fragebögen getroffen. Ebenfalls kontaktiert wurden die zwei Medizinischen Zentren für Erwachsene mit Behinderungen im Stadtgebiet München, das Medizinische Versorgungszentrum der Pfennigparade, das an eine stationäre Wohneinrichtung angegliedert ist und MmB versorgt, sowie die zwei Therapiepraxen der Pfennigparade.

Die Kontaktaufnahme über Einrichtungen der Behindertenhilfe, Verteiler und Netzwerke ermöglichte eine anonyme und datenschutzkonforme Befragung. Tab. 2 zeigt, wie viele Einrichtungen kontaktiert und wie viele Papierfragebögen versendet wurden.

**Table:** **Tab. 2** Kontaktierte Einrichtungen und Versand der Fragebögen.

Kontaktierte Einrichtungen der Behindertenhilfe				
	Stationäre Einrichtungen	Wohngruppen	MZEB ^a^ /MVZ ^b^ /Praxis	gesamt
Anzahl Einrichtungen	22	37	5	**64**
Anzahl Plätze	926	299	k.A.	**1225**
**Anzahl der versendeten Fragebögen**				
Alltagssprache	111	140	25	276
leichte Sprache	141	179	25	345
gesamt	252	319	50	**621**

^a^ MZEB=Medizinisches Zentrum für Erwachsene mit Behinderungen; ^b^ MVZ=Medizinisches Versorgungszentrum

Die Befragung lief vom 08.08.2024 bis 31.10.2024. Online-Rückmeldungen wurden in einem Datensatz beim Statistischen Amt München gesammelt. Papierfragebögen wurden in vorfrankierten Rücksendeumschlägen an das Gesundheitsreferat der Landeshauptstadt München zurückgeschickt.

### Datenanalysen

Es wurden deskriptive Analysen zu absoluten und relativen Häufigkeiten durchgeführt. Variablen, die Ausprägungen mit geringer Häufigkeit aufwiesen, wurden durch Zusammenfassen von Antwortoptionen umkodiert (siehe Tab. 1 Markierung (d)). Da es keinen Indikator für die „hausärztliche Versorgung“ gab und diese nicht durch eine einzelne Variable dargestellt werden konnte, wurden in der Analyse mehrere Variablen herangezogen, um die hausärztliche Versorgung abzubilden. In Tab. 1 ist markiert (b), welche Variablen hierfür verwendet wurden. Anschließend wurde anhand bivariater Analysen die hausärztliche Versorgung in Abhängigkeit der Variablen *Alter, Geschlecht, Art der Beeinträchtigung* und *Wohnform* untersucht, um Unterschiede in der Versorgung zwischen Personengruppen zu identifizieren. Die Variable *Art der Beeinträchtigung* wies aufgrund der Möglichkeit für Mehrfachnennungen keine Unabhängigkeit der Beobachtungen auf. Um sie dennoch für bivariate Analysen verwenden zu können, wurde sie wie folgt umkodiert: körperliche- und Sinnesbeeinträchtigungen, geistige oder seelisch-psychische Beeinträchtigungen, chronische Krankheit, mehrfache Beeinträchtigungen.

Die Variable *Notwendigkeit Hausbesuche* wurde ebenfalls anhand bivariater Analysen untersucht. Hierfür wurde die Variable durch Umkodierung dichotomisiert. In Tab. 1 sind die Variablen markiert (c), die für diese Analysen verwendet wurden. Aufgrund des explorativen Studienansatzes wurde keine Korrektur für multiples Testen durchgeführt.

Um mögliche Einschränkungen beim Zugang zur Versorgung zu identifizieren, wurden anhand einer logistischen Regressionsanalyse Prädiktoren für das Auftreten von Schwierigkeiten bei der Suche nach einer Hausärztin/einem Hausarzt (Frage 3) ermittelt. Zunächst wurden Variablen identifiziert, die für das Regressionsmodell als sinnvoll erachtet wurden. Ungeeignete Variablen und Variablen, die Merkmale mit geringen Ausprägungen aufwiesen, wurden ausgeschlossen. Mithilfe von Chi-Quadrat-Tests wurden Variablen vorselektiert; als Signifikanzniveau wurde ein p-Wert von≤0,05 festgelegt. Die in das Modell einzuschließenden Variablen wurden anhand des Spearman Korrelationskoeffizienten auf Multikollinearität getestet. Als Kriterium für die Reihenfolge des Einschlusses der signifikanten unabhängigen Variablen wurde die Höhe des bivariaten Zusammenhangs mit der abhängigen Variable gewählt. In ein erstes Modell wurden nur Variablen aufgenommen, die einen signifikanten (p≤0,05) und substanziellen Erklärungsbeitrag (Verbesserung Nagelkerke R ^2^ ) zum Modell lieferten. Einem zweiten, adjustierten Modell wurden forced-in Variablen ( *Krankenversicherung, Art der Beeinträchtigung, Alter* und *Geschlecht* ) hinzugefügt. Zur Überprüfung der Multikollinearität des finalen Modells wurde der Varianzinflationsfaktor für die in das Modell eingeschlossenen Variablen berechnet.

Die Auswertungen erfolgten mit der Statistik Software SPSS Version 29.0.2.0 (20).

## Ergebnisse

Insgesamt nahmen 306 Personen teil, davon 77% (n=235) an der Online-Befragung und 23% (n=71) an der schriftlichen Befragung. Fragebögen in leichter Sprache verwendeten in der Online-Befragung 11% der Teilnehmenden (n=34) und in der schriftlichen Befragung 32% (n=23). Insgesamt 51% (n=153) der Befragten füllten den Fragebogen selbstständig aus; 49% (n=148) erhielten Unterstützung.

Tabelle 3 zeigt die Angaben zur Person (Angaben zu Geschlecht, Alter, Wohnform, Krankenversicherung, Art der Beeinträchtigung, Grad der Behinderung und Merkzeichen). 54% (n=165) der Befragten waren Frauen. Der höchste Anteil der Teilnehmenden waren 40-55 Jahre (27%, n=82) alt, wohnte selbstständig (38%, n=112) und war gesetzlich versichert (93%, n=278). Es wurden alle abgefragten Arten der Beeinträchtigungen angegeben, mit 59% (n=177) am häufigsten körperliche Beeinträchtigungen. Einen Grad der Behinderung hatten 91% (n=277) der Befragten, davon 60% (n=162) einen Grad der Behinderung von 100. Merkzeichen sind im Schwerbehindertenausweis eingetragene Buchstaben, die die Art der Beeinträchtigung spezifizieren oder Berechtigungen enthalten 11 . 72% (n=196) der Teilnehmenden vorliegender Studie hatten eingetragene Merkzeichen. Mit jeweils 63% wurden die Merkzeichen „gehbehindert“ (n=122) und „Begleitung erforderlich“ (n=121) am häufigsten angegeben.

**Table:** **Tab. 3** Beschreibung der Teilnehmenden: Geschlecht, Alter, Wohnform, Krankenversicherung, Art der Beeinträchtigung, GdB ^c^ , Merkzeichen.

weiblich	165		53,9	
männlich	129		42,2	
divers	1		0,3	
keine Angabe	11		3,6	
**Alter** (n=304, m.d. ^a^ n=2)	**n**		**%**	
18-25 Jahre	40		13,2	
26-39 Jahre	81		26,6	
40-55 Jahre	82		27,0	
56-69 Jahre	80		26,3	
70 Jahre und älter	21		6,9	
**Wohnform** (n=297, m.d. ^a^ n=9)	**n**		**%**	
selbstständig in Wohnung oder Haus	112		37,7	
mit Assistenz in Wohnung oder Haus	12		4,0	
bei Familie/Eltern	39		13,1	
ambulantes betreutes Wohnen	28		9,4	
Wohngruppe	40		13,5	
stationäre Einrichtung/Wohnheim	66		22,2	
**Krankenversicherung** (n=299, m.d ^a^ n=7)	**n**		**%**	
gesetzlich	278		93,0	
privat	21		7,0	
**Art der Beeinträchtigung** (n=298, m.d. ^a^ n=8) ^**b**^	**ja**		**nein**	
	**n**	**%**	**n**	**%**
körperliche Beeinträchtigung	177	59,4	121	40,6
Schwerhörigkeit/ Gehörlosigkeit/ Taubheit	33	11,1	265	88,9
Lernbeeinträchtigung	46	15,4	252	84,6
Blindheit/ starke Sehbeeinträchtigung	40	13,4	258	86,6
geistige Beeinträchtigung	97	32,6	201	67,4
seelische oder psychische Beeinträchtigung	77	25,8	221	74,2
chronische Krankheit	139	46,6	159	53,4
**GdB**^**c**^ (n=304, m.d. ^a^ n=2)	**n**		**%**	
ja	277		91,1	
nein	17		5,6	
ich bin mir nicht sicher	10		3,3	
**Höhe des GdB**^**c, d**^ (n=272, m.d. ^a^ n=34)	**n**		**%**	
20	0		0,0	
30	14		5,1	
40	8		2,9	
50	33		12,1	
60	17		6,3	
70	10		3,7	
80	16		5,9	
90	12		4,4	
100	162		59,6	
**Merkzeichen erhalten**^**d**^ (n=273, m.d. ^a^ n=33)	**n**		**%**	
ja	196		71,8	
nein	61		22,3	
ich bin mir nicht sicher	16		5,9	
**Angaben zu den Merkzeichen**^**b, d, e**^ (n=193, m.d. ^a^ n=113)	**ja**		**nein**	
	**n**	**%**	**n**	**%**
aG ^f^	75	38,9	118	61,1
G ^g^	122	63,2	71	36,8
H ^h^	105	54,4	88	45,6
Bl ^i^	24	12,4	169	87,6
B ^j^	121	62,7	72	37,3
Gl ^k^	18	9,3	175	90,7
TBl ^l^	2	1,0	191	99,0
RF ^m^	60	31,1	133	68,9

^a^ Missing Data; ^b^ Mehrfachantworten waren möglich; ^c^ Grad der Behinderung; ^d^ nur Befragte, die einen GdB haben; ^e^ nur Befragte, die Merkzeichen bekommen haben; ^f^ außergewöhnlich gehbehindert; ^g^ gehbehindert; ^h^ hilflos; ^i^ blind; ^j^ Begleitung erforderlich; ^k^ gehörlos; ^l^ taubblind; ^m^ Rundfunkbeitragsbefreiung oder -ermäßigung

### Inanspruchnahme hausärztlicher Versorgung

Insgesamt gaben 95% (n=291) der Befragten an, eine Hausärztin/einen Hausarzt zu haben. Davon benötigen 69% (n=193) die Hausärztin/den Hausarzt aufgrund ihrer Beeinträchtigungen. Fast alle Befragten (93%, n=272) waren im letzten halben Jahr bei der Hausärztin/dem Hausarzt; die Hälfte (55%, n=162) innerhalb des letzten Monats. Am häufigsten gaben die Befragten an, innerhalb der letzten 12 Monate 3-4x (n=97, 35%) in hausärztlicher Behandlung gewesen zu sein. 16% (n=45) gaben an, mehr als 10-mal dort gewesen zu sein.

### Barrieren beim Hausarztbesuch

Insgesamt 40% (n=122) der Befragten gab an, Schwierigkeiten bei der Suche nach einer Hausärztin/einem Hausarzt zu haben oder gehabt zu haben. Bereits von einer Hausärztin/einem Hausarzt abgewiesen wurden 30% (n=91); ein Patient*innen-Aufnahmestopp der Arztpraxis war bei 45% (n=41) der Grund hierfür. Die eigenen Beeinträchtigungen nannten 33% (n=30) der Befragten als Grund, fehlende Barrierefreiheit 22% (n=20).

Die Hälfte der Befragten (50%, n=152) berichtete beim Hausarztbesuch eingeschränkt zu sein. Davon gaben 76% (n=116) an, aufgrund ihrer Beeinträchtigungen und 38% (n=57) aufgrund fehlender Barrierefreiheit eingeschränkt zu sein. Eine fehlende Begleitperson zum Arztbesuch gaben 17% (n=26) als Grund an. Bei 70% befand sich die hausärztliche Praxis in der Nähe des Wohnortes (n=218) und war gut mit öffentlichen Verkehrsmitteln zu erreichen (n=212). In Abb. 1 sind die Angaben der Befragten zur baulichen, sprachlichen und sozialen Barrierefreiheit in der Hausarztpraxis dargestellt.

**Abb. 1:**
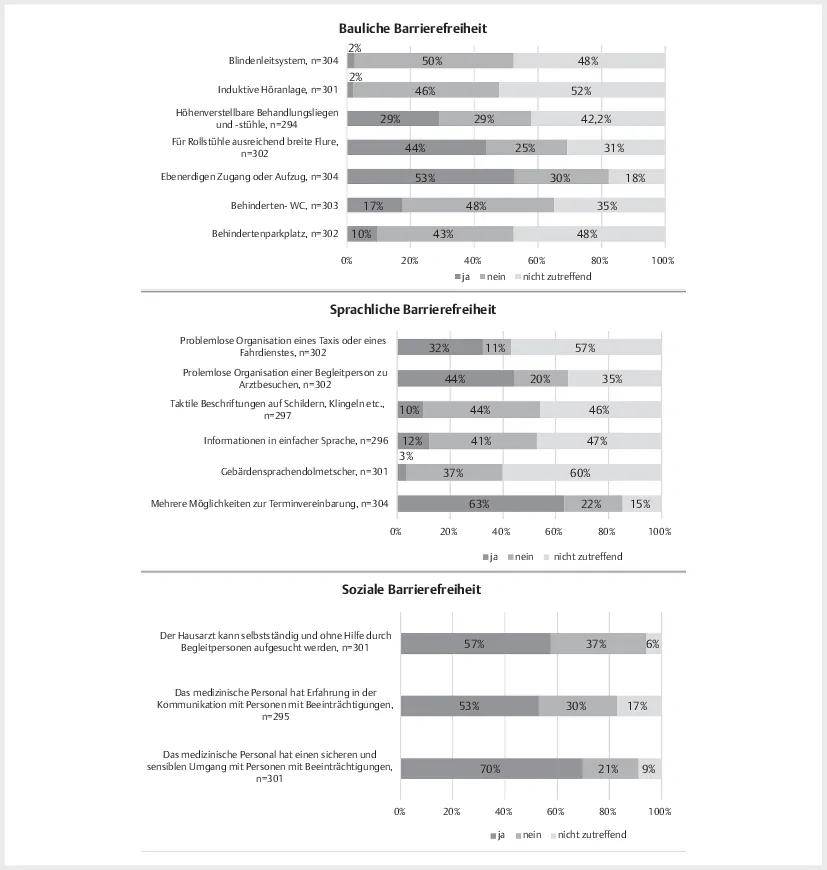
Angaben zu baulicher, sprachlicher und sozialer Barrierefreiheit.

### Zufriedenheit mit Hausärztin/Hausarzt, Praxispersonal und medizinischer Behandlung

Über 80% der Befragten waren mit ihrer Hausärztin/ihrem Hausarzt (85%, n=300), dem Praxispersonal (86%, n=292) und der medizinischen Behandlung (83%, n=302) sehr oder eher zufrieden ( Abb. 2 ).

**Abb. 2:**
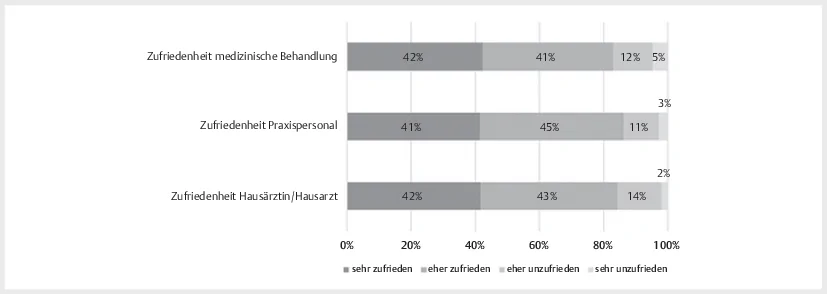
Zufriedenheit mit Hausärztin/Hausarzt, Praxispersonal und medizinischer Behandlung.

### Unterschiede in der hausärztlichen Versorgung in Abhängigkeit von Alter, Geschlecht, Wohnform und Art der Beeinträchtigung

Es konnten geschlechts- und wohnformbedingte Unterschiede in der Versorgung sowie Unterschiede je nach Art der Beeinträchtigung festgestellt werden.

Alle signifikanten Unterschiede in Abhängigkeit von *Geschlecht* , *Wohnform* und *Art der Beeinträchtigung* sind in Tabelle A1 dargestellt. Keine signifikanten Unterschiede konnten festgestellt werden für die Versorgung in Abhängigkeit von *Alter (* siehe Tabelle A2).

Die unten genannten Prozentangaben beziehen sich auf die Gesamtzahl der unabhängigen Variable, um Unterschiede im Bezug zur Versorgung darzustellen.

Frauen suchten sich häufiger ihre Hausärztin/ihren Hausarzt selbst aus (80%, n=124) als Männer (54%, n=65), wurden häufiger von einer Hausärztin/einem Hausarzt abgewiesen (36%, n=59 vs. 23%, n=30) und waren in den letzten 12 Monaten häufiger mehr als 10-mal in hausärztlicher Behandlung (18%, n=28 vs. 10%, n=11).

MmB, die stationär wohnten, benötigten im Vergleich zu selbstständig oder ambulant lebenden MmB häufiger Hausbesuche (52%, n=34 vs. 29%, n=36 bzw. 30%, n=32), wurden seltener von einer Hausärztin/einem Hausarzt abgewiesen (11%, n=7 vs. 39%, n=48 bzw. 30%, n=32) und nahmen in den letzten 12 Monaten häufiger mehr als 10-mal hausärztliche Termine in Anspruch (25%, n=16 vs. 16%, n=19 bzw. 11%, n=10). Im Vergleich zu MmB, die selbstständig oder ambulant wohnten, suchten sich MmB, die stationär wohnten, ihre Hausärztin/ihren Hausarzt deutlich seltener selbst aus (22%, n=14 vs. 94%, n=112 bzw. 65%, n=64). Menschen mit mehrfachen Beeinträchtigungen brauchten häufiger Hausbesuche (42%, n=73) im Vergleich zu Personen mit Körper- und Sinnesbeeinträchtigungen (33%, n=23), geistigen oder seelisch psychischen Beeinträchtigungen (12%, n=5) oder chronischen Erkrankungen (29%, n=4).

Auch benötigten sie ihre Hausärztin/ihren Hausarzt am häufigsten aufgrund ihrer Beeinträchtigungen (76%, n=120 vs. Körper- und Sinnesbeeinträchtigungen 65%, n=43, geistig oder seelisch psychischen Beeinträchtigungen 51%, n=18, chronischen Erkrankungen 71%, n=10). Menschen mit geistigen oder seelisch-psychischen Beeinträchtigungen suchten sich am seltensten (46%, n=17) ihre Hausärztin/ihren Hausarzt selbst aus.

### Versorgung bei Bedarf nach Hausbesuchen

Ein Viertel der Befragten (25%, n=75) gab an, Hausbesuche zu benötigen, während 10% (n=31) angaben, zwar Hausbesuche zu benötigen, diese jedoch nicht zu erhalten. Zudem berichteten 35% (n=106), dass ihre Hausärztin/ihr Hausarzt keine Hausbesuche anbiete. Diejenigen, die Hausbesuche benötigten, hatten häufiger Schwierigkeiten bei der Suche nach einer Hausärztin/einem Hausarzt (64%, n=62 vs. 32%, n=60) und wurden häufiger abgewiesen (50%, n=44 vs. 25%, n=47). Sie hatten häufiger im letzten Monat einen Termin bei der Hausärztin/dem Hausarzt (68%, n=72 vs. 45%, n=89)und nahmen im letzten Jahr häufiger mehr als zehnmal hausärztliche Hilfe in Anspruch (21%, n=26 vs. 9%, n=18).

Am häufigsten benötigten Personen mit mehrfachen Beeinträchtigungen Hausbesuche (70%, n=73), gefolgt von Personen mit körperlichen oder Sinnesbeeinträchtigungen (22%, n=23), Personen mit geistigen oder seelisch-psychischen Beeinträchtigungen (5%, n=5 sowie Personen mit chronischen Beeinträchtigungen (4%, n=4)). Personen, die keine Hausbesuche benötigten, wohnten am häufigsten selbstständig (45%, n=87), verglichen zu Personen, die ambulant (39%, n=75) oder stationär (17%, n=32) wohnten. In Tabelle A3 und A4 sind die Ergebnisse für die Versorgung in Abhängigkeit von der Notwendigkeit von Hausbesuchen dargestellt.

### Vorhersage für die Wahrscheinlichkeit, Schwierigkeiten bei der Suche nach einer Hausärztin/einem Hausarzt zu haben

Es wurden 13 Variablen für die Aufnahme in das Regressionsmodell identifiziert und in den Analysen berücksichtigt. Das initiale Modell enthielt die folgenden drei Variablen: *Notwendigkeit Hausbesuche, vom Hausarzt abgewiesen, Einschränkungen beim Hausarztbesuch* (Nagelkerke R ^2^ =0,545) (vgl. Tab. 4 ).

**Table:** **Tab. 4** Ergebnisse der multivariaten logistischen Regression mit Zielvariable „Hatten oder haben Sie Schwierigkeiten bei der Suche nach einem Hausarzt?“. Initiales Modell: drei Variablen; Adjustiertes Modell: Einschluss der forced-in Variablen. Anmerkung: in den Fragebögen wurde auf gendergerechte Sprache verzichtet, weshalb auch in dieser Tabelle aufgrund inhaltlicher Richtigkeit nicht gegendert wird.

	Initiales Modell		Adjustiertes Modell	
**Prädiktoren** in ( ) Referenz	**OR** (KI* unterer Wert; oberer Wert)	**p**	**OR** (KI* unterer Wert; oberer Wert)	**p**
**Vom Hausarzt abgewiesen** (ja)	**18,945** (8,896; 40,347)	<0,001	**18,461** (8,223; 41,448)	<0,001
**Einschränkungen beim Hausarztbesuch** (ja)	**5,397** (2,735; 10,653)	<0,001	**7,076** (3,238; 15,463)	<0,001
**Notwendigkeit Hausbesuche** (ja)	**2,428** (1,227; 4,803)	0,011	**2,197** (1,026; 4,705)	0,043
**Geschlecht (weiblich)**				
Männlich	–	–	1,691 (0,821; 3,482)	0,154
Andere	–	–	2,682 (0,292; 24,665)	0,383
**Versicherung (gesetzlich)**				
Privat versichert	–	–	1,276 (0,264; 6,156)	0,762
**Alter (18-25 Jahre)**				
26-39 Jahre	–	–	1,413 (0,418; 4,773)	0,578
40-55 Jahre	-	–	1,168 (0,346; 3,945	0,803
56-69 Jahre	-	–	0,651 (0,185; 2,289)	0,503
70 Jahre und älter	-	–	2,467 (0,424; 14,349)	0,315
**Art d. Beeinträchtigung (mehrfache Beeinträchtigungen)**				
Körper- und Sinnesbeeinträchtigungen	–	–	0,865 (0,388; 1,925)	0,722
Geistige oder seelisch-psychische Beeinträchtigungen	–	–	1,153 (0,333; 3,999)	0,822
Chronische Beeinträchtigungen	–		3,202 (0,451; 22,723)	0,245
Konstante	0,046	<0,001	0,029	<0,001
Anzahl der in das Regressionsmodell eingeschlossenen Fälle	**279**		**263**	
Nagelkerke R ^2^	**0,545**		**0,575**	

* Konfidenzintervall

Die vier Variablen *Krankenversicherung, Art der Beeinträchtigung, Alter* und *Geschlecht* waren im initialen Modell nicht signifikant. Sie wurden aufgrund ihrer inhaltlichen Relevanz einem zweiten, adjustierten Modell als forced-in Variablen hinzugefügt, was zu einer Verbesserung der Modellgüte führte (Nagelkerke R ^2^ =0,575) (vgl. Tab. 4 ).

Das adjustierte Modell zeigt, dass die Wahrscheinlichkeit, dass eine Person Schwierigkeiten bei der Suche nach einer Hausärztin/einem Hausarzt hat oder hatte, 18,5-mal höher war, wenn sie angab, bereits einmal von einer Hausärztin/einem Hausarzt abgewiesen worden zu sein. Zudem hatten Personen, die angaben, eingeschränkt beim Hausarztbesuch zu sein, eine 7-mal höhere Wahrscheinlichkeit Schwierigkeiten bei der Suche nach einer Hausärztin/einem Hausarzt zu haben oder gehabt zu haben. Personen, die Hausbesuche benötigten, hatten eine 2,2-mal höhere Wahrscheinlichkeit Schwierigkeiten bei der Suche zu haben oder gehabt zu haben, im Vergleich zu denen, die keine Hausbesuche benötigten.

## Diskussion

Die Studie analysiert die hausärztliche Versorgung von MmB in München aus Sicht der MmB. Fast alle Befragten gaben an eine Hausärztin/einen Hausarzt zu haben und eine große Mehrheit war mit ärztlichem und Praxispersonal sowie medizinischer Behandlung zufrieden. Eine repräsentative Versichertenbefragung des GKV-Spitzenverbands aus dem Jahr 2024 ergab auch bei Menschen ohne Behinderungen hohe Zufriedenheitswerte mit der Hausärztin/dem Hausarzt. So gaben im Jahr 2024 68% der Befragten an, mit der Hausärztin/dem Hausarzt vollkommen oder sehr zufrieden zu sein. Allerdings gaben auch 13% eine fehlende Kompetenz des ärztlichen Personals und unzureichende Aufklärung über Behandlungen an 12 . Auch in vorliegender Studie wurde trotz hoher Zufriedenheitswerte von Unsicherheit und fehlender Erfahrung des medizinischen Personals im Umgang mit MmB berichtet. Eine Studie aus dem Jahr 2023, in der Menschen mit kognitiver Beeinträchtigung , die in Werkstätten für behinderte Menschen in Nordrhein-Westfalen arbeiteten, zu ihrer Zufriedenheit mit Ärzt*innen befragt wurden, ergab ähnliche Werte 13 . Das Bundesministerium für Familie, Senioren, Frauen und Jugend veröffentlichte 2013 eine Studie zur Lebenssituation von Frauen mit Behinderungen und Beeinträchtigungen in Deutschland, in der Frauen in Haushalten und Einrichtungen via Vor-Ort-Befragung an ausgewählten Standorten befragt wurden 14 . Auch in dieser Studie gaben etwa 80% der Befragten an, zufrieden mit der gesundheitlichen Versorgung zu sein 14 . Allerdings wurde sowohl in der Studie aus dem Jahr 2023, als auch in der Studie aus dem Jahr 2013 ebenfalls von unsensiblem Umgang und kritischen Erfahrungen in ärztlichen Praxen berichtet 5 13 14 . Diese Ergebnisse unterstreichen, dass trotz hoher Zufriedenheitswerte die Bedürfnisse von MmB nicht immer adäquat berücksichtigt werden. Dem könnte durch Schulungen und Fortbildungen von medizinischem Personal entgegengewirkt werden. Neben umfassenden Weiterbildungen sollten insbesondere auch zeitschonende, kompakte Fortbildungen für alle medizinischen Berufe angeboten werden.

Knapp die Hälfte der Befragten gab an, Schwierigkeiten bei der Suche nach einer Hausärztin/einem Hausarzt zu haben oder gehabt zu haben; ein Drittel wurde abgewiesen und die Hälfte war beim Hausarztbesuch eingeschränkt. Häufig wurden eigene Beeinträchtigungen als Grund hierfür genannt. Ein weiterer entscheidender Grund war fehlende Barrierefreiheit. Insbesondere Maßnahmen der Barrierefreiheit bei Sinnesbeeinträchtigungen durch z. B. Blindenleitsysteme, induktive Höranlagen und Gebärdensprachdolmetscher waren nur in wenigen Praxen vorhanden. Deutlich häufiger gab es breite, rollstuhlgeeignete Flure sowie einen ebenerdigen Zugang bzw. Aufzug zur Praxis. Barrierefreiheit für mobilitätseingeschränkte Personen gewinnt mittlerweile an Aufmerksamkeit, Anforderungen für Personen mit Sinnes- oder Lernbeeinträchtigungen sowie geistigen und seelisch oder psychischen Beeinträchtigungen dagegen weniger 3 . So erfüllen laut Bundesteilhabebericht etwa 21% der vertragsärztlichen Praxen nach eigenen Angaben die Kriterien der uneingeschränkten Barrierefreiheit für mobilitätseingeschränkte Personen, nur etwa 7% hingegen haben Orientierungshilfen für sehbehinderte Menschen 3 . Bis 2024 galt eine Praxis laut Bundesarztregister der Kassenärztlichen Bundesvereinigung als uneingeschränkt barrierefrei, wenn ein ebenerdiger, rollstuhlgerechter Zugang, breite Türen und breite Bewegungsflächen vorhanden waren 3 . Im Juli 2024 veröffentlichte die Kassenärztliche Bundesvereinigung eine neue Richtlinie zur Barrierefreiheit 15 und betonte, dass Anforderungen unterschiedlicher Beeinträchtigungen zu berücksichtigen sind. Die Versichertenbefragung des GKV-Spitzenverbandes aus dem Jahr 2024 ergab, dass zwar die Kontaktrate zu Hausärzt*innen als hoch angegeben wurde und auch Wartezeiten für hausärztliche Termine als zufriedenstellend eingeschätzt wurden, es jedoch Schwierigkeiten beim Zugang zur Versorgung aufgrund von Mängel bei Terminservicestellen, nicht ausreichende Öffnungszeiten der ärztlichen Praxen sowie einer verschlechterten Erreichbarkeit gab 12 . Die Autor*innen schlussfolgern, dass diese Herausforderungen MmB zusätzlich zu den oben genannten Schwierigkeiten bei der Suche nach ärztlichen Praxen betreffen.

Fast alle in der vorliegenden Studie befragten MmB waren innerhalb der letzten 12 Monate bei der Hausärztin/dem Hausarzt und die meisten gaben an, drei- bis viermal dort gewesen zu sein. Auch Menschen ohne Behinderungen gingen laut einer Versichertenbefragung der Kassenärztlichen Bundesvereinigung am häufigsten drei- bis fünfmal jährlich zur Hausärztin/zum Hausarzt 16 . Allerdings zeigt sich, dass die in unserer Studie befragten MmB im Vergleich zu Menschen ohne Beeinträchtigungen der Versichertenbefragung der Kassenärztlichen Bundesvereinigung deutlich häufiger hausärztliche Termine wahrnehmen. Auch die Ergebnisse der GEDA-Studie des Robert Koch Instituts aus dem Jahr 2014/2015 zeigen eine höhere Inanspruchnahme von ambulanten ärztlichen Leistungen von MmB im Vergleich zu Menschen ohne Beeinträchtigungen 4 .

In unserer Studie zeigte sich, dass insbesondere MmB, die Hausbesuche benötigten und mehrfache Beeinträchtigungen aufwiesen, einen erhöhten Bedarf nach hausärztlichen Terminen hatten. Auch war bei der Inanspruchnahme von hausärztlichen Terminen ein Geschlechtsunterschied zu erkennen. Frauen nahmen häufiger als Männer mehr als zehn Mal in den letzten 12 Monaten hausärztliche Termine in Anspruch und suchten sich auch häufiger ihre Hausärztin/ihren Hausarzt selbst aus. Auch in der GEDA-Studie zeigten Frauen mit Beeinträchtigungen eine höhere Inanspruchnahme ambulanter ärztlicher Leistungen im Vergleich zu Männern mit Beeinträchtigungen 4 . Generell gelten Frauen als gesundheitsbewusster und sind eher dazu geneigt, ärztliche Hilfe in Anspruch zu nehmen 17 . Die höhere Inanspruchnahme hausärztlicher Termine von MmB, insbesondere bei Bedarf nach Hausbesuchen und bei mehrfachen Beeinträchtigungen sowie die nur bedingten Geschlechtsunterschiede könnten darauf zurückgeführt werden, dass MmB aufgrund ihrer gesundheitlichen Herausforderungen und komplexen Krankheitsbilder generell eine umfassendere Versorgung benötigen 4 6 . Dies verdeutlicht die Notwendigkeit einer bedarfsgerechten gesundheitlichen Versorgung für MmB.

Dass die Inanspruchnahme von Hausbesuchen für MmB erschwert ist, wird unter anderem dadurch deutlich, dass, obwohl viele MmB in unserer Studie Hausbesuche benötigten, häufig berichtet wurde, dass Hausärzt*innen keine Hausbesuche anbieten würden. Zudem wurden MmB, die Hausbesuche benötigten, häufiger von einer hausärztlichen Praxis abgewiesen. Laut Bundesmantelvertrag sind Hausärzt*innen zu regelmäßigen Hausbesuchen bei Patient*innen, die bettlägerig, gebrechlich oder pflegebedürftig sind, verpflichtet 9 . Die Zahl der Hausbesuche ist seit 2009 deutlich gesunken 18 , obwohl mit zunehmender Alterung unserer Bevölkerung und der dadurch bedingten Zunahme insbesondere chronischer Erkrankungen 19 von einem steigenden Bedarf nach ärztlicher Versorgung zuhause auszugehen ist. Der deutsche Hausärztinnen- und Hausärzteverband kritisiert die geringen Vergütungen bei Hausbesuchen und fordert Anpassungen, um Hausbesuche wieder wirtschaftlich zu machen 19 .

Laut unserer Studie benötigten insbesondere MmB in stationären Einrichtungen häufig Hausbesuche. Ihre Versorgung in den Einrichtungen wird häufig durch engagierte niedergelassene Hausärzt*innen sichergestellt, die Kooperationsverträge mit Einrichtungen der Behindertenhilfe abgeschlossen haben 20 . Unserer Studie zeigte, dass stationär wohnende MmB im Vergleich zu selbstständig oder ambulant wohnenden MmB sich deutlich seltener ihre Hausärztin/ihren Hausarzt selbst aussuchten. Diese Ergebnisse sind ein Hinweis darauf, dass die o.g. Kooperationsverträge dazu führen könnten, dass Personen in stationären Einrichtungen sich ihre Hausärztin/ihren Hausarzt nicht selbst aussuchen. In §76 des SGB V ist das Recht auf freie Ärztin-/Arztwahl aller gesetzlich versicherten Personen geregelt 21 . Die Kooperationsverträge, aber auch fehlende Barrierefreiheit und Abweisungen bedeuten für MmB ein eingeschränktes Recht auf freie Ärztin-/Arztwahl. Am häufigsten eingeschränkt in der freien Ärztin/ Arztwahl waren geistig oder seelisch-psychisch beeinträchtigte Befragte unserer Studie. Diese sind häufig für den Zugang zur Versorgung auf Angehörige oder betreuende Personen angewiesen, die die Organisation und Begleitung der ärztlichen Besuche übernehmen 13 . Die Ergebnisse verdeutlichen, dass das Recht auf freie Ärztin-/Arztwahl für MmB eingeschränkt ist, allerdings sind hierfür auch Maßnahmen bzw. Unterstützung zur Sicherstellung der Versorgung von Menschen in Einrichtungen der Behindertenhilfe verantwortlich.

Die o.g. Kooperationsverträge führen zu einer temporären Sicherstellung der Versorgung von MmB in Einrichtungen der Behindertenhilfe. Dem Gesundheitsreferat der Landeshauptstadt München ist durch den Austausch mit Trägern der Behindertenhilfe bekannt, dass es bei Renteneintritt der kooperierenden Ärztin/des kooperierenden Arztes allerdings erhebliche Unsicherheiten und Schwierigkeiten bei der Organisation einer Nachfolgerin/eines Nachfolgers gibt. Dass diese Bedenken durchaus berechtigt sind, zeigt das Durchschnittsalter deutscher Hausärzt*innen, das im Jahr 2023 bei 55,2 Jahren lag 22 . Etwa 37% der deutschen Hausärzt*innen sind über 60 Jahre alt und knapp 70% sind über 50 Jahre alt 22 .

Mithilfe der Regressionsanalyse wurden Prädiktoren für die Wahrscheinlichkeit, Schwierigkeiten bei der Suche nach einer Hausärztin/einem Hausarzt zu haben oder gehabt zu haben, ermittelt. Diese war signifikant erhöht, wenn Personen bereits von einer Praxis abgewiesen wurden, angaben beim Hausarztbesuch eingeschränkt zu sein und Hausbesuche benötigten. Wichtig ist daher, Gründe für Abweisungen und Einschränkungen zu identifizieren und den Zugang zur Versorgung bei Bedarf nach Hausbesuchen zu verbessen. Gründe für Abweisungen in der vorliegenden Studie waren das Vorhandensein von Beeinträchtigungen, fehlende Barrierefreiheit und Aufnahmestopps der Arztpraxis. Einschränkungen beim Hausarztbesuch traten in unserer Studie ebenfalls aufgrund fehlender Barrierefreiheit und den eigenen Beeinträchtigungen sowie fehlenden Begleitpersonen auf. Die Ergebnisse der Regressionsanalyse unterstreichen daher die Notwendigkeit, Barrierefreiheit zu verbessern, Angebote für Schulungen für medizinisches Personal im Umgang mit MmB auszuweiten und den Zugang zur Versorgung bei Bedarf nach Hausbesuchen zu erleichtern.

Zur gesundheitlichen Versorgung von MmB gibt es bisher in Deutschland nur wenige Daten 3 4 . Die für diese Studie verwendeten Angaben zur Evidenz basieren auf deutsche Publikationen, da sich die Angaben der Befragten zur ambulanten hausärztlichen Versorgung in unserer Studie auf das deutsche Gesundheitssystem beziehen und eine internationale Vergleichbarkeit aufgrund der unterschiedlichen Gesundheitssysteme schwierig ist. Aufgrund der mangelnden Datenlage wurden auch Studien herangezogen, in denen andere Zielgruppen, z. B. Frauen mit Behinderungen oder Menschen mit kognitiven Beeinträchtigungen , befragt wurde. Hierdurch konnten Gemeinsamkeiten gefunden werden, die zur Übertragbarkeit von Aussagen auf verschiedene Zielgruppen beitragen könnten.

Vorhandene Literatur zur gesundheitlichen Versorgung von MmB, wie der Mikrozensus der Schwerbehindertenstatistik, Teilhabeberichte der Bundesregierung oder Berichte zur Gesundheit in Deutschland basieren auf verschiedenen Datenquellen und schließen teilweise nur MmB ein, die amtlich anerkannte Behinderungen haben 4 . MmB sind eine heterogene Personengruppe, die verschiedene Bedürfnisse und Gesundheitszustände aufweist 4 . Für diese Studie gab es daher keine Ausschlusskriterien zur Art oder Schwere der Behinderung, wodurch Personen mit einem breiten Spektrum an Beeinträchtigungen erreicht wurden. Dieses Vorgehen wurde auch gewählt, da Personen nicht erst ab einem Grad der Einschränkung oder Schwere einer Erkrankung als beeinträchtigt zählen, sondern dann, wenn ihre wirksame und gleichberechtigte Teilhabe an der Gesellschaft langfristig eingeschränkt ist 2 .

Das Angebot des Fragebogens in leichter Sprache sowie die Möglichkeit zur Online- und schriftlichen Teilnahme an der Befragung sollte möglichst vielen MmB die Teilnahme an der Studie erleichtern. Beide Optionen wurden von den Befragten in Anspruch genommen. Auch die Möglichkeit zur Unterstützung beim Ausfüllen des Fragebogens wurde stark genutzt und ermöglichte MmB, die nicht über die Fähigkeit verfügten, einen Fragebogen selbstständig auszufüllen, die Teilnahme an der Befragung.

Einige Konfidenzintervalle der Prädiktoren der logistischen Regressionsanalyse sind sehr groß, was auf eine Unsicherheit bzgl. der Aussage dieser Prädiktoren hinweisen könnte. Diese Unsicherheit kann bei der Variable *Geschlecht* in der Kategorie „andere“ auf die geringe Anzahl an Beobachtungen zurückgeführt werden. Personen, die sich entweder nicht zu ihrem Geschlecht äußern wollten oder sich nicht dem weiblichen oder männlichen Geschlecht zugeordnet fühlten, wurden zur Erhaltung der Diversität nicht randomisiert einem Geschlecht zugeordnet, sondern in der Kategorie „andere“ erfasst und in den Analysen berücksichtigt. Auch das große Konfidenzintervall der Variable *vom Hausarzt abgewiesen* weist auf die Unsicherheit der Beobachtung hin, allerdings trägt die Variable maßgeblich zur Modellgüte bei, was sich in einer Sensitivitätsanalyse durch Berechnung des Modells ohne die Variable *vom Hausarzt abgewiesen* zeigte (Nagelkerke R ^2^ =0,356 vgl. mit R ^2^ =0,575). Vermutlich sind der hohe Schätzer und das große Konfidenzintervall auf die geringe Trennschärfe der Zielvariable *Schwierigkeiten bei der Suche* und der Variable *vom Hausarzt abgewiesen* zurückzuführen. Darauf weist auch die hohe Korrelation der zwei Variablen hin (Spearman Korrelationskoeffizient, r=0,591).

## Limitationen und Begrenzungen der Untersuchung

Diese Studie beschränkt sich auf die ambulante hausärztliche Versorgung von MmB im Stadtgebiet München. Die Inanspruchnahme und Herausforderungen der fachärztlichen Versorgung von MmB sowie größer angelegte Untersuchungen sowohl im urbanen als auch im ländlichen Raum, sind zur Betrachtung der ambulanten Versorgungssituation von MmB von großer Relevanz und können Gegenstand weiterer Studien werden. Diese würden auch zur Verbesserung der Datenlage zur medizinischen Versorgung von MmB in Deutschlang maßgeblich beitragen. Für Befragungen in anderen Städten und/oder Bundesländern wäre ein evidenzbasierter, erprobter und etablierter Fragebogen sinnvoll. Dadurch könnten einheitliche Daten erhoben werden und Ergebnisse miteinander verglichen werden.

Das Design dieser Studie ließ keine repräsentative Studienpopulation zu. Daher besteht die Möglichkeit einer Stichprobenverzerrung. Voraussetzung zur Teilnahme war ein Wohnsitz in München oder eine/n in München praktizierende/n Hausärztin/Hausarzt. Dadurch wurde versucht, die Versorgung von MmB in München durch Münchner Hausärzt*innen zu erheben. Allerdings wurde der Wohnort nicht abgefragt, da sich bei der Pilotierung zeigte, dass eine genaue Angabe des Wohnorts für MmB Schwierigkeiten verursachte. Genaue Angaben zu hausärztlichen Praxen wurden aus Datenschutzgründen nicht abgefragt. Es ist daher unklar, welche MmB erreicht wurden, weshalb die Ergebnisse dieser Studie keine Generalisierbarkeit zulassen. Durch die Streuung der Informationen zur Befragung über Netzwerke von und für MmB sowie über Soziale Medien ist die Anzahl der erreichten MmB unbekannt, wodurch keine Aussage zur Rücklaufquote getroffen werden kann. Die Kontaktaufnahme über Einrichtungen der Behindertenhilfe sowie die Streuung des Fragebogens über Akteure der Behindertenhilfe wurde aus zeitlichen und Datenschutzgründen gewählt.

Für die Variable *Art der Beeinträchtigung* waren Mehrfachantworten möglich, weshalb sie keine unabhängigen Beobachtungen aufwies und für bivariate und multivariate Analysen nicht geeignet war. Die Variable wurde entsprechend umkodiert und aufgrund geringer Randhäufigkeiten mussten Beeinträchtigungsformen zusammengefasst werden. Dadurch wurden die Vielfältigkeit und Heterogenität der verschiedenen Beeinträchtigungsformen in diesen Analysen eingeschränkt. Für eine genauere Betrachtung der Bedürfnisse der verschiedenen Beeinträchtigungsformen ist eine größere Stichprobe notwendig.

Eine weitere Limitation dieser Studie könnte sein, dass die Ergebnisse auf subjektiven Einschätzungen der MmB beruhen. In der Literatur werden Befragungen von Menschen mit kognitiven Beeinträchtigungen und mehrfachen Behinderungen teilweise als kritisch angesehen, da diese zu sozial erwünschten Antworten neigen und Antworten inkonsistent sein können 23 . Die hohen Zufriedenheitswerte, die im Widerspruch zu den Angaben zu unsicherem und unsensiblem Umgang stehen, könnten ein Hinweis hierauf sein. Allerdings deckt sich diese Beobachtung mit Ergebnissen anderer Studien 13 14 .

Für unsere Studie befragten wir MmB zu ihrer hausärztlichen Versorgungssituation und betrachteten dadurch lediglich ihre Sicht auf die Versorgung. Um ein umfassenderes Bild von der Versorgung von MmB zu erhalten und Herausforderungen und Bedürfnisse anderer an der Versorgung Beteiligter zu identifizieren, müssten weitere Perspektiven beleuchtet werden. Sinnvoll wären daher auch Befragungen von Hausärzt*innen, Praxispersonal, betreuenden Personen und Angehörigen zur hausärztlichen Versorgung von MmB.

## Fazit und Ausblick

Diese Studie untersuchte die hausärztliche Versorgung von MmB in München. Es konnte keine Unterversorgung aus der Perspektive der MmB im Bereich der hausärztlichen Versorgung festgestellt werden, jedoch traten Probleme beim Zugang zur Versorgung auf und Bedürfnisse von MmB wurden nicht immer adäquat berücksichtigt. Zur Verbesserung der Versorgung sollten Barrierefreiheit ausgebaut und medizinisches Personal im Umgang mit MmB geschult werden. Dabei sind sämtliche Beeinträchtigungsformen einzubeziehen. Aufgrund komplexer Gesundheitslagen benötigen MmB längere Behandlungszeiten und häufiger Hausbesuche. Künftige Studien sollten auch fachärztliche und stationäre Versorgungsangebote in unterschiedlichen Regionen einbeziehen.

## Zusätzliches Material

Zusätzliches Material Zusätzliches Material
