# Supplementary material for: Primary care for people with disabilities in Munich: results of a cross-sectional study
Source: Gesundheitswesen. 2026 Jun 22;88(11):641–54. [Article in German] doi: 10.1055/a-2833-6852 (PMC13747670; doi:10.1055/a-2833-6852)
Supplement: Supplementary file 1 [file 10-1055-a-2833-6852-2025-07-2303-oa.pdf]

ZUSATZMATERIAL:

Tabelle A1: Bivariate Analysen zur Versorgung in Abhängigkeit von Geschlecht, Wohnform und Art der Beeinträchtigung: ***signifikante Ergebnisse.*** *Anmerkung: in den Fragebögen wurde auf gendergerechte Sprache verzichtet, weshalb auch in dieser Tabelle aufgrund inhaltlicher Richtigkeit nicht gegendert wird.*

		Geschlecht			
		weiblich		männlich	
Hausarzt selbst ausgesucht ** p<0,001, n=276	ja	124	80,0%	65	53,7%
	nein	31	20,0%	56	46,3%
Termin letzte 12 Monate p=0,007, n=266	0x	0	0,0%	1	0,9%
	1-2x	38	25,0%	26	22,8%
	3-4x	48	31,6%	48	42,1%
	5-10x	38	25,0%	28	24,6%
	>10x	28	18,4%	11	9,6%
vom Hausarzt abgewiesen p=0,044, n=293	ja	59	35,8%	30	23,4%
	nein	106	64,2%	98	76,6%

		Wohnform					
		selbstständig		ambulant		stationär	
Notwendigkeit Hausbesuche p=0,004, n=296	ja	36	29,3%	32	29,9%	34	51,5%
	nein	87	70,7%	75	70,1%	32	48,5%
Termin letzte 12 Monate p=0,003, n=276	0x	1	0,8%	2	2,1%	5	7,8%
	1-2x	27	22,9%	30	31,9%	7	10,9%
	3-4x	37	31,4%	34	36,2%	25	39,1%
	5-10x	34	28,8%	18	19,1%	11	17,2%
	>10x	19	16,1%	10	10,6%	16	25,0%
Hausarzt selbst ausgesucht p<0,001, n=281	ja	112	94,1%	64	64,6%	14	22,2%
	nein	7	5,9%	35	35,4%	49	77,8%
vom Hausarzt abgewiesen p<0,001, n=296	ja	48	38,7%	32	30,2%	7	10,6%
	nein	76	61,3%	74	69,8%	59	89,4%

		Art der Beeinträchtigung							
		Körper- und Sinnesbeeinträchtigungen		Geistige oder seelisch psychische Beeinträchtigungen		Chronische Krankheit		Mehrfache Beeinträchtigungen	
Notwendigkeit Hausbesuche p=0,004, n=297	ja	23	33,3%	5	12,2%	4	28,6%	73	42,2%
	nein	46	66,7%	36	87,8%	10	71,4%	100	57,8%
Hausarzt aufgrund von Beeinträchtigungen p=0,033, n=274	ja	43	65,2%	18	51,4%	10	71,4%	120	75,5%
	nein	23	34,8%	17	48,6%	4	28,6%	39	24,5%
Hausarzt selbst ausgesucht p<0,001, n=280	ja	54	80,6%	17	45,9%	12	92,3%	107	65,6%
	nein	13	19,4%	20	54,1%	1	7,7%	56	34,4%

Einschränkungen beim Hausarztbesuch p<0,002, n=294	ja	33	48,5%	11	26,8%	5	35,7%	100	58,5%
	nein	35	51,5%	30	73,2%	9	64,3%	71	41,5%
Letzter Termin p<0,001, n=295	innerhalb des letzten	35	51,5%	12	29,3%	10	71,4%	102	59,3%
	im letzten halben Jahr	26	38,2%	18	43,9%	3	21,4%	61	35,5%
	vor weniger als einem	2	2,9%	3	7,3%	1	7,1%	6	3,5%
	vor mehr als einem Jahr	1	1,5%	4	9,8%	0	0,0%	0	0,0%
	vor mehr als zwei Jahren	1	1,5%	2	4,9%	0	0,0%	0	0,0%
	weiß ich nicht	3	4,4%	2	4,9%	0	0,0%	3	1,7%
Termin letzte 12 Monate p<0,001, n=278	0x	0	0,0%	1	3,0%	0	0,0%	0	0,0%
	1-2x	22	35,5%	12	36,4%	3	21,4%	28	16,6%
	3-4x	23	37,1%	14	42,4%	2	14,3%	56	33,1%
	5-10x	14	22,6%	3	9,1%	2	14,3%	47	27,8%
	mehr als 10x	3	4,8%	1	3,0%	7	50,0%	33	19,5%
	weiß ich nicht	0	0,0%	2	6,1%	0	0,0%	5	3,0%

Tabelle A2: Bivariate Analysen zur Versorgung in Abhängigkeit von Geschlecht, Wohnform und Art der Beeinträchtigung: ***nicht signifikante*** *Ergebnisse*

		Geschlecht			
		weiblich		männlich	
Notwendigkeit Hausbesuche p=0,213, n=293	ja	55	33,3%	44	53,7%
	nein	110	66,7%	84	46,3%
Hausarzt aufgrund von Beeinträchtigungen p=0,085, n=272	ja	98	63,6%	87	73,7%
	nein	56	36,4%	31	26,3%
Letzter Termin p=0,082, n=291	Innerhalb d. letzten Monats	89	54,3%	64	50,4%
	Im letzten halben Jahr	61	37,2%	49	38,6%
	Vor weniger als einem Jahr	7	4,3%	5	3,9%
	Vor mehr als einem Jahr	2	1,2%	3	2,4%
	Vor mehr als zwei Jahren	0	0,0%	2	1,6%
	Weiß ich nicht	5	3,0%	4	3,1%
Schwierigkeiten bei der Suche p=0,151, n=275	ja	74	48,1%	44	36,4%
	nein	80	51,9%	77	63,6%

Hausarzt vorhanden p=0,766, n=294	ja	158	95,8%	122	94,6%
	nein	7	4,2%	7	5,4%
Einschränkungen beim Hausarztbesuch P=0,066, n=290	ja	80	48,8%	62	49,2%
	nein	84	51,2%	64	50,8%

		**Wohnform**					
		selbstständig		ambulant		stationär	
Schwierigkeiten bei der Suche p=0,162, n=175	ja	58	47,5%	42	42,0%	17	32,1%
	nein	64	52,5%	58	58,0%	36	67,9%
Letzter Termin p=0,099, n=294	Innerhalb d. letzten Monats	69	56,6%	45	42,5%	43	65,2%
	Im letzten halben Jahr	45	36,9%	43	40,6%	20	30,3%
	Vor weniger als einem Jahr	4	3,3%	7	6,6%	1	1,5%
	Vor mehr als einem Jahr	1	0,8%	4	3,8%	0	0,0%
	Vor mehr als zwei Jahren	1	0,8%	2	1,9%	0	0,0%
	Weiß ich nicht	2	1,6%	5	4,7%	2	3,0%
Hausarzt aufgrund von Beeinträchtigungen p=0,335, n=274	ja	78	66,1%	62	66,7%	48	76,2%
	nein	40	33,9%	31	33,3%	15	23,8%
Hausarzt vorhanden p=283, n=297	ja	119	96,0%	100	93,5%	65	98,5%
	nein	5	4,0%	7	6,5%	1	1,5%
Einschränkungen beim Hausarztbesuch P=0,306, n=294	ja	56	45,5%	55	51,9%	37	56,9%
	nein	67	54,5%	51	48,1%	28	43,1%

		**Art der Beeinträchtigung**							
		Körper- und Sinnesbeeinträchtigungen		Geistige oder seelisch psychische Beeinträchtigungen		Chronische Krankheit		Mehrfache Beeinträchtigungen	
Schwierigkeiten bei der Suche p=0,111, n=276	ja	31	46,3%	11	29,7%	3	21,4%	72	45,6%
	nein	36	53,7%	26	70,3%	11	78,6%	86	54,4%
Hausarzt vorhanden p=0,316, n=298	ja	67	97,1%	37	90,2%	14	100,0%	166	95,4%
	nein	2	2,9%	4	9,8%	0	0,0%	8	4,6%
vom Hausarzt abgewiesen p=0,599, n=297	ja	24	34,8%	10	24,4%	3	21,4%	51	29,5%
	nein	45	65,2%	31	75,6%	11	78,6%	122	70,5%

		Alter									
		18-25 Jahre		26-39 Jahre		40-55 Jahre		56-69 Jahre		70 und älter	
Notwendigkeit Hausbesuche p=0,315, n=303	ja	9	22,5%	26	32,1%	28	34,1%	33	41,8%	8	38,1%
	nein	31	77,5%	55	67,9%	54	65,9%	46	58,2%	13	61,9%
Schwierigkeiten bei der Suche p=0,403, n=283	ja	17	44,7%	33	42,9%	34	45,3%	33	44,6%	4	21,1%
	nein	21	55,3%	44	57,1%	41	54,7%	41	55,4%	15	78,9%
Hausarzt aufgrund von Beeinträchtigungen p=0,711, n=281	ja	25	69,4%	52	72,2%	52	66,7%	49	64,5%	15	78,9%
	nein	11	30,6%	20	27,8%	26	33,3%	27	35,5%	4	21,1%
Hausarzt aufgrund von Beeinträchtigungen p=0,701. n=304	ja	37	92,5%	76	93,8%	79	96,3%	78	97,5	20	95,2%
	nein	3	7,5%	5	6,2%	3	3,7%	2	2,5%	1	4,8%
Hausarzt selbst ausgesucht p=0,071, n=286	ja	24	64,9%	44	57,9%	54	70,1%	54	71,1%	18	90,0%
	nein	13	35,1%	32	42,1%	23	29,9%	22	28,9%	2	10,0%
Letzter Termin p=0,711, n=301	Innerhalb d. letzten Monats	16	40,0%	39	48,1%	46	56,8%	47	58,8%	13	68,4%
	Im letzten halben Jahr	18	45,0%	32	39,5%	25	30,9%	30	37,5%	5	26,3%
	Vor weniger als einem Jahr	2	5,0%	5	6,2%	5	6,2%	0	0,0%	1	5,3%
	Vor mehr als einem Jahr	1	2,5%	2	2,5%	2	2,5%	0	0,0%	0	0,0%
	Vor mehr als zwei Jahren	1	2,5%	1	1,2%	0	0,0%	1	1,3%	0	0,0%
	Weiß ich nicht	2	5,0%	2	2,5%	3	3,7%	2	2,5%	0	0,0%
Termin letzte 12 Monate p=0,259, n=283	0x	1	2,8%	0	0,0%	0	0,0%	0	0,0%	0	0,0%
	1-2x	12	33,3%	22	28,9%	17	22,7%	11	14,3%	5	26,3%
	3-4x	11	30,6%	25	32,9%	25	33,3%	30	39,0%	6	31,6%
	5-10x	6	16,7%	12	15,8%	16	21,3%	26	33,8%	6	31,6%
	mehr als 10x	5	13,9%	14	18,4%	15	20,0%	8	10,4%	2	10,5%
	weiß ich nicht	1	2,8%	3	3,9%	2	2,7%	2	2,6%	0	0,0%
vom Hausarzt abgewiesen p=0,187, n=303	ja	11	27,5%	28	35,0%	28	34,1%	22	27,5%	2	9,5%
	nein	29	72,5%	52	65,0%	54	65,9%	58	72,5%	19	90,5%
Einschränkungen beim Hausarztbesuch p=0,121, n=300	ja	22	56,4%	44	55,0%	43	53,7%	30	37,5%	12	57,1%
	nein	17	43,6%	36	45,0%	37	46,3%	50	62,5%	9	42,9%

Tabelle A3: Bivariate Analysen zur hausärztlichen Versorgung bei Notwendigkeit von Hausbesuchen: ***signifikante Ergebnisse.*** *Anmerkung: in den Fragebögen wurde auf gendergerechte Sprache verzichtet, weshalb auch in dieser Tabelle aufgrund inhaltlicher Richtigkeit nicht gegendert wird. Anmerkung 2: Für bivariate Analysen wurde die Variable „Notwendigkeit Hausbesuche" dichotomisiert. Hierfür wurde die Antwortoption „ich bräuchte Hausbesuche, bekomme aber keine" der Antwortkategorie „ja" zugeordnet.*

	**Notwendigkeit Hausbesuche**			
	ja	%	nein	%
**Schwierigkeiten bei der Suche, p<0,001, n=283**				
ja	62	64,6	60	32,1
nein	34	35,4	127	67,9
**vom Hausarzt abgewiesen, p<0,001, n=273**				
ja	44	50,0	47	25,4
nein	44	50,0	138	74,6
**letzter Termin, p=0,004, n=302**				
innerhalb des letzten Monats	72	67,9	89	45,4
im letzten halben Jahr	30	28,3	80	40,8
vor weniger als einem Jahr	1	0,9	12	6,1
vor mehr als einem Jahr	0	0,0	5	2,6
vor mehr als zwei Jahren	1	0,9	2	1,0
weiß ich nicht	2	1,9	8	4,1
**Termin letzte 12 Monate, p=0,002, n=274**				
1-2x	15	11,8	52	24,4
3-4x	30	45,7	67	49,3
5-10x	28	22,0	38	17,8
>10x	26	20,5	18	8,5
**Art der Beeinträchtigung, p=0,004, n=297**				
Körper- und Sinnesbeeinträchtigung	23	21,9	46	24,0
Geistige und seelisch-psychische Beeinträchtigung	5	4,8	36	18,8
Chronische Beeinträchtigung	4	3,8	10	5,2
Mehrfache Beeinträchtigung	73	69,5	100	52,0
**Wohnform, p=0,004, n=296**				
Selbstständig	36	35,3	87	44,8
Ambulant	32	31,4	75	38,7
Stationär	34	33,3	32	16,5

Tabelle A4: Bivariate Analysen zur hausärztlichen Versorgung bei Notwendigkeit von Hausbesuchen: ***nicht signifikante Ergebnisse****. Anmerkung: in den Fragebögen wurde auf gendergerechte Sprache verzichtet, weshalb auch in dieser Tabelle aufgrund inhaltlicher Richtigkeit nicht gegendert wird. Anmerkung 2: Für bivariate Analysen wurde die Variable „Notwendigkeit Hausbesuche" dichotomisiert. Hierfür wurde die Antwortoption „ich bräuchte Hausbesuche, bekomme aber keine" der Antwortkategorie „ja" zugeordnet.*

	**Notwendigkeit Hausbesuche**			
	ja	%	nein	%
**Alter, p=0,315, n=303**				
18-25 Jahre	9	22,5	31	77,5
26-39 Jahre	26	32,1	55	67,9
40-55 Jahre	28	34,1	54	65,9
56-69 Jahre	33	41,8	46	58,2
70 Jahre und älter	8	38,1	13	61,9
